# Somatostatin Receptor 1 and 5 Double Knockout Mice Mimic Neurochemical Changes of Huntington's Disease Transgenic Mice

**DOI:** 10.1371/journal.pone.0024467

**Published:** 2011-09-02

**Authors:** Padmesh S. Rajput, Geetanjali Kharmate, Michael Norman, Shi-He Liu, Bhagavatula R. Sastry, Charles F. Brunicardi, Ujendra Kumar

**Affiliations:** 1 Division of Pharmacology and Toxicology, Faculty of Pharmaceutical Sciences, The University of British Columbia, Vancouver, British Columbia, Canada; 2 Department of Surgery, Baylor College of Medicine, Houston, Texas, United States of America; 3 Neuroscience Research Laboratory, Department of Anesthesiology, Pharmacology and Therapeutics, The University of British Columbia, Vancouver, British Columbia, Canada; City of Hope National Medical Center and Beckman Research Institute, United States of America

## Abstract

**Background:**

Selective degeneration of medium spiny neurons and preservation of medium sized aspiny interneurons in striatum has been implicated in excitotoxicity and pathophysiology of Huntington's disease (HD). However, the molecular mechanism for the selective sparing of medium sized aspiny neurons and vulnerability of projection neurons is still elusive. The pathological characteristic of HD is an extensive reduction of the striatal mass, affecting caudate putamen. Somatostatin (SST) positive neurons are selectively spared in HD and Quinolinic acid/N-methyl-D-aspartic acid induced excitotoxicity, mimic the model of HD. SST plays neuroprotective role in excitotoxicity and the biological effects of SST are mediated by five somatostatin receptor subtypes (SSTR1-5).

**Methods and Findings:**

To delineate subtype selective biological responses we have here investigated changes in SSTR1 and 5 double knockout mice brain and compared with HD transgenic mouse model (R6/2). Our study revealed significant loss of dopamine and cAMP regulated phosphoprotein of 32 kDa (DARPP-32) and comparable changes in SST, N-methyl-D-aspartic acid receptors subtypes, calbindin and brain nitric oxide synthase expression as well as in key signaling proteins including calpain, phospho-extracellular-signal-regulated kinases1/2, synapsin-IIa, protein kinase C-α and calcineurin in SSTR1/5^−/−^ and R6/2 mice. Conversely, the expression of somatostatin receptor subtypes, enkephalin and phosphatidylinositol 3-kinases were strain specific. SSTR1/5 appears to be important in regulating NMDARs, DARPP-32 and signaling molecules in similar fashion as seen in HD transgenic mice.

**Conclusions:**

This is the first comprehensive description of disease related changes upon ablation of G- protein coupled receptor gene. Our results indicate that SST and SSTRs might play an important role in regulation of neurodegeneration and targeting this pathway can provide a novel insight in understanding the pathophysiology of Huntington's disease.

## Introduction

Huntington's disease (HD) is an inherited autosomal dominant neurodegenerative disorder caused by mutation in the *huntingtin (Htt)* gene and characterized by progressive chorea and impaired cognitive function [1], [2]. Genetic abnormality of expanded polyglutamine repeat sequence is confined in the coding region of a gene IT15 located on chromosome 4 encoding the Htt protein [3], [4]. The length of CAG repeat is one of the factors that plays an important role in the onset of HD symptoms [5], [6]. Pathological characteristics of the disease are the intranuclear inclusion of mutated Htt and neostriatum atrophy and gliosis.

In addition to genetic mutation and histopathological hallmarks, the critical determinant of HD is the degeneration of medium size spiny neurons (MSNs) expressing γ-aminobutyric acid (γ-GABA), N-methyl-D-aspartic acid receptors (NMDARs) and dopamine and cAMP regulated phosphoprotein of 32 kDa (DARPP-32). In contrast, in striatum, a subset of neuronal population consisting of medium sized aspiny interneurons positive to somatostatin (SST), neuropeptide Y (NPY) and nicotinamide adenine dinucleotide phosphate-diaphorase (NADPH-d)/brain nitric oxide synthase (bNOS) are selectively spared [7], [8]. In addition, the expression of calbindin D-28K is increased in HD patients, transgenic mouse models and quinolinic acid (QUIN)-induced excitotoxicity [9], [10], [11]. Activation of NMDARs in striatum mimics the pathological, neurochemical and behavioral changes of HD [12]. Furthermore, the analysis of HD patient's postmortem brain reveals the degeneration of NMDAR-positive neurons and association with the pathogenesis in HD [13], [14]. NMDARs are composed of two subunits of NR1 and two subunits of NR2A, NR2B or NR2C [15], [16]. Previous studies have shown enhanced NMDAR-mediated toxicity in cells expressing mutated Htt as well as in HD mouse models [17], [18]. Recently, the functional importance of NMDARs emerged from a study describing the role of NMDAR antagonist memantine to block the nuclear inclusion of Htt in yeast artificial chromosome (YAC) mice [19]. These data suggest that NMDARs play an important role in HD and may contribute to neuronal loss.

NMDA replicate the neurophathological features of HD and have been used as models of the disease [20], [21]. In the striatum of experimental mice, medium-sized aspiny interneurons expressing SST, NPY and NADPH-d/bNOS are selectively resistant to QUIN-induced excitotoxicity. Similarly, such interneurons are relatively well spared observations in the brains of HD patients [19], [21], [22], [23], [24], [25], [26]. Previous studies have also shown increased SST secretion and gene expression in HD brain and NMDA/QUIN-induced excitotoxicity [21], [23], [27]. In support of the selective preservation of interneurons, it was argued that these neurons lack NMDARs [28]. In contrast, several recent studies have shown the presence of NMDARs in SST/NPY/NOS positive neurons in striatum of rat brain and cultured striatal neurons [4], [29], [30]. Most importantly, we have recently shown that immunoblockade of SST by using antisense oligoneucleotides and immunoneutralization of released SST by using SST specific antibodies potentiate neuronal loss in QUIN/NMDA-induced excitotoxicity in cultured striatal neurons, including NPY, NADPH-d and bNOS positive neurons [31]. Furthermore, selective sparing of SST positive neurons in bNOS knockout mice suggests that the presence of SST is essential for the survival of interneurons [32].

The presence of SST in the central and peripheral nervous system is associated with several physiological functions, which are attributed to different receptor subtypes, namely somatostatin receptor 1–5 (SSTR1-5), which are members of G-protein coupled receptor (GPCR) family. All five SSTR subtypes display overlapping distribution in different parts of brain and importantly couple to G_i_ protein and inhibit cAMP in a pertussis toxin sensitive manner. SSTRs are involved in the regulation of ion channels; inhibition of Ca^2+^ and activation of K^+^ channels involved in the release of several neurotransmitters and modulation of neurotransmission [33]. These functional properties of SSTR subtypes can be further enhanced by interaction with members of their own family as well as other GPCRs, including dopamine and opioid receptors via heterodimerization [34], [35], [36], [37]. Widespread distribution of SSTRs in CNS is involved in various neurological diseases such as Huntington's disease (HD), Alzheimer's disease (AD), Parkinson's disease (PD), epilepsy, HIV encephalitis, dementia and psychiatric disorders, including schizophrenia [22], [38], [39], [40], [41], [42]. These studies cumulatively suggest the critical and pivotal role of SSTR subtypes in neurodegenerative diseases.

We recently observed that the knock-down of SSTR 1 and SSTR 5 using antisense oligonucleotides accelerated neuronal death upon NMDA treatment in cultured striatal neurons (unpublished observations). Accordingly, in an attempt to elucidate the possible functions of SSTR1 and 5, the present study was undertaken to determine the expression of NMDARs, DARPP-32, calbindin, bNOS/SST and SSTRs in striatum of R6/2 and SSTR1/5^−/−^mice. In addition, we also studied the downstream signaling cascades including calcineurin, calpain, PKC-α, ERK1/2, synapsin-IIa and enkephalin associated in the process of neurodegeneration in HD pathology as well as in experimental models of the disease. In the present study, for the first time, we describe that the SSTR1/5 complex is a critical regulator of NMDARs, DARPP-32 and downstream signaling cascades normally seen in R6/2 transgenic mice. Importantly, this study revealed that SSTR1/5^−/−^ mice mimic neuro-and biochemical changes of presymptomatic HD transgenic mice.

## Results

### Decreased DARPP-32 expression and increased Calbindin D-28K expression in striatum of SSTR1/5^−/−^ mice and R6/2 mice brain

**Figure 1:**
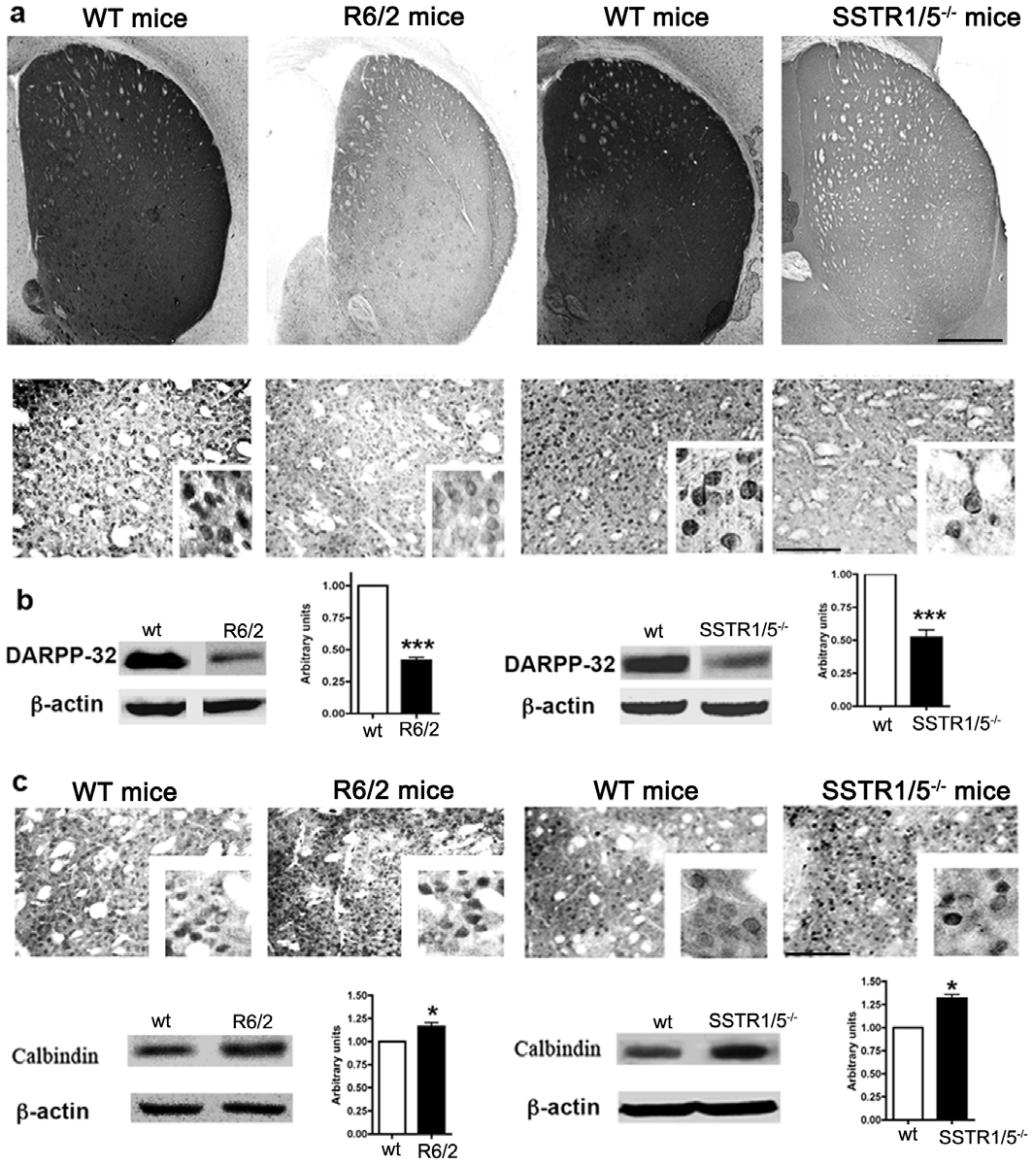
Representative low- and high-magnification photomicrogrpahs depicting comparative distribution of DARPP-32 immunoreactivity in striatum of R6/2 and SSTR1/5^−/−^ mice. DARPP-32 like immunoreactivity was lost in striatum of R6/2 and SSTR1/5^−/−^ mice, respectively (**a**). Comparable decreased expression of DARPP-32 (32 kDa) in R6/2 and SSTR1/5^−/−^ mice was also seen in Western blot analysis (**b**). Calbindin like immunoreactivity was found to be increased in both R6/2 and SSTR1/5^−/−^ mice compared to respective *wt* mice **(c upper panel)**. Increased calbindin (28 kDa) expression was further confirmed with Western blot analysis **(c bottom panel)**. Data presented as mean ± SD for Western blot analysis (SSTR1/5^−/−^, n = 5 and R6/2 mice, n = 3) in comparison to *wt* mice brain,***P<0.001 and *P<0.05. Scale bar = 150 µm and 20 µm upper and bottom panel **a**, 20 µm panel **c** and 5 µm for inset.

Neuronal population in striatum is largely composed of medium sized projection neurons, which are positive for DARPP-32 and constitute 85% of total neuronal population. Several previous studies have shown significant loss of DARPP-32 like immunoreactivity in HD patients and/or in experimental models of disease [43], [44]. Accordingly, here we examined the DARPP-32 like immunoreactivity using immunohistochemical and Western blot analysis in R6/2 and SSTR1/5^−/−^ as well as respective *wt* mice brains. As illustrated in Figure 1a, consistent with previous studies, a marked decrease in signal intensity of DARPP-32 was observed in R6/2 when compared to *wt* mice. Like R6/2 mice, a similar decrease in DARPP-32 like immunoreactivity was observed in SSTR1/5^−/−^ when compared to *wt* mice. DARPP-32 like immunoreactivity is mostly expressed as cytoplasmic protein. Consistent with our immunocytochemistry data, Western blot analysis showed a significant decrease in DARPP-32 expression in comparison to *wt* in the brains of both R6/2 and SSTR1/5^−/−^ mice (Figure 1b).

Similar to DARPP-32, calbindin D-28K immunoreactivity was detected in the striatum and has been used as an index of MSN. As illustrated in Figure 1c, increased expression of calbindin-like immunoreactivity was observed in R6/2 and SSTR1/5^−/−^ when compared to *wt* mice. Western blot analysis showed consistent results as seen in immunohistochemistry with increased expression of calbindin in striatum of R6/2 and SSTR1/5^−/−^ mice (Figure 1c).

### SST and bNOS positive neurons are selectively spared in striatum of R6/2 and SSTR1/5^−/−^ mice

**Figure 2:**
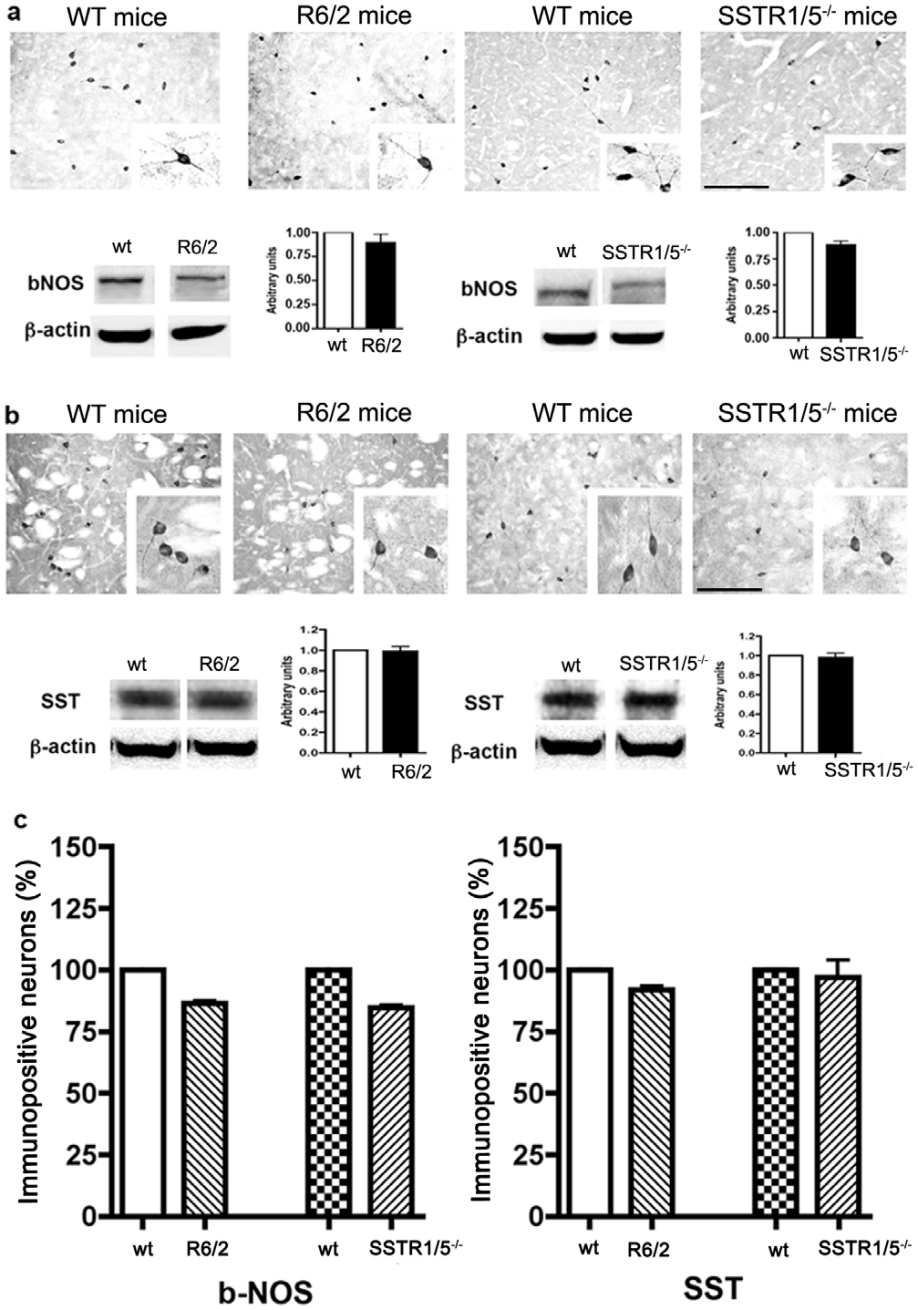
Comparative immunohistochemical localization of bNOS and SST in striatum of R6/2 and SSTR1/5^−/−^ mice. The expression level of bNOS **(a upper panel)** was comparable in both strains. Furthermore, Western blot analysis revealed no significant changes in bNOS (160 kDa) expression **(a bottom panel)**. As shown in upper panel b expression of SST in both the strains was comparable. Western blot analysis reveals no change in expression of SST (28 kDa) in both the strains **(b bottom panel)** when compared to respective *wt*. Histogram represent quantitative analysis of bNOS and SST positive neurons (**c**). Data presented as mean ± SD (SSTR1/5^−/−^, n = 5 and R6/2 mice, n = 3) in comparison to *wt* mice brain. Scale bar = 20 µm panel **a and b** and 5 µm for inset.

Previous studies have shown that medium-sized aspiny interneurons positive for bNOS/ NADPH-d also co-express SST and NPY and are selectively preserved in HD patients as well as experimental models of disease [7], [8]. Accordingly, we determined the expression of bNOS and SST-positive neurons in striatum of R6/2 and SSTR1/5^−/−^ mice brain. As shown in Figure 2a, bNOS immunoreactivity was observed in sparsely distributed neurons all over the striatum with arborizing neuronal processes without discernable changes in R6/2 and SSTR1/5^−/−^ mice when compared to *wt* mice. In addition, Western blot analysis also revealed no significant changes in bNOS expression levels in R6/2 and SSTR1/5^−/−^ mice in comparison to their counterpart *wt*. As shown in Figure 2b, the neuronal population exhibiting SST like immunoreactivity was comparable in R6/2 and SSTR1/5^−/−^ mice using immunohistochemistry and Western blot analysis. As shown in Figure 2c, further quantitative analysis revealed comparable changes in bNOS and SST positive neurons in R6/2 and SSTR1/5^−/−^ mice as well as in comparison to *wt*. R6/2 transgenic mice exhibited 14 and 8% loss of bNOS and SST positive neurons, respectively. Conversely, SSTR1/5^−/−^ mice showed 15 and 3% loss of bNOS and SST positive neurons respectively, not significantly different from *wt* mice.

### Expression of NMDA receptor subtypes in the striatum of SSTR1/5^−/−^ and R6/2 mice

**Figure 3:**
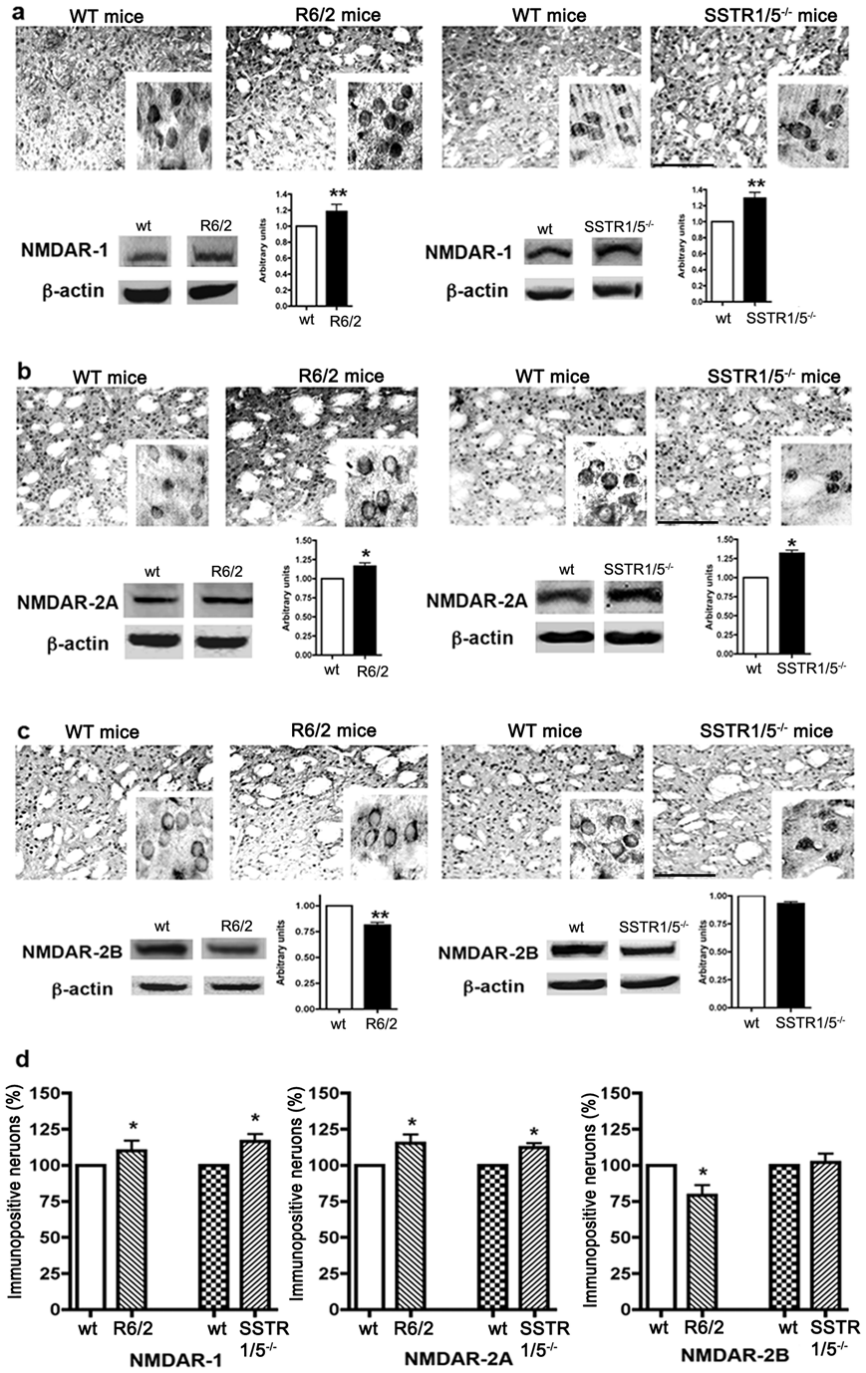
Representative photomicrographs illustrating distribution of NMDAR subtypes expression in striatum of R6/2 and SSTR1/5^−/−^ mice. Comprarable distribution pattern of NR1 (**a**), NR2A (**b**) and NR2B (**c**) was seen between R6/2 mice and SSTR1/5^−/−^ mice. Quantitaive analysis of receptor immunoreactivity was accomplished using Western blot analysis. Note the increased expression of NR1 (120 kDa) immunoreactivity **(a upper panel)** and expression by Western blot **(a lower panel)** in R6/2 and SSTR1/5^−/−^ mice. NR2A (180 kDa) expression was increased in R6/2 and SSTR1/5^−/−^ mice (**b**). Decreased expression of NR2B (190 kDa) was seen in R6/2 mice whereas no significant changes in SSTR1/5^−/−^ mice when compared to *wt* mice (**c**). Note cytosolic accumulation of NR2A and NR2B in SSTR1/5^−/−^ mice and membrane expression in R6/2 mice. Percentage changes in NMDARs positive neurons in R6/2, SSTR1/5^−/−^ mice and *wt* mice are shown in panel (**d**). Data presented as mean ± SD for Western blot analysis (SSTR1/5^−/−^, n = 5 and R6/2 mice, n = 3) in comparison to *wt* mice brain, *P<0.05, ** P<0.01. Scale bar = 20 µm panel **a–c** and 5 µm for inset.

The role of activated NMDARs in degeneration of medium spiny neurons in HD as well as in excitotoxicity is indisputable. Several previous studies have shown the activation of NMDAR subtypes in the brain of R6/2 transgenic mice [18]. We sought to determine whether R6/2 and SSTR1/5^−/−^ mice exhibit comparable cellular distribution pattern in NMDAR subunits and determined the expression levels of NR1, NR2A and NR2B using immunohistochemistry and Western blot analysis. As shown in Figure 3a, in *wt* mice brain NR1-like immunoreactivity in striatal neurons was confined to the cell membranes, as well as, intracellularly. Additionally, NR1-like immunoreactivity was also seen in nerve fibers. In comparison to *wt*, R6/2 mice brain displayed strong NR1 immunoreactivity that was restricted to cell membrane. Consistent with immunohistochemistry, increased receptor expression was observed in striatal tissue lysate prepared from R6/2 mice in comparison to *wt*. Similar pattern of NR-1 immunoreactivity was observed in SSTR1/5^−/−^ mice in comparison to *wt* using immunohistochemistry and Western blot analysis (Figure 3a). Similar to the NR1 expression, NR2A subunit expression was increased in R6/2 and SSTR1/5^−/−^ mice when compared to respective *wt* (Figure 3b). Interestingly, NR2A immunoreactivity expressed at cell surface in R6/2 mice in comparison to SSTR1/5^−/−^ mice where receptor immunoreactivity was markedly intracellular. Western blot analysis revealed enhanced NR2A expression in SSTR1/5^−/−^ mice in comparison to R6/2 mice as well as respective *wt*. In comparison to NR1 or NR2A expression, R6/2 and SSTR1/5^−/−^ mice exhibited the loss of NR2B immunoreactivity. The loss of NR2B was to a greater extent in R6/2 brain than SSTR1/5^−/−^ mice (Figure 3c). The distributional pattern of NR2B-like immunoreactivity was significantly different in R6/2 and SSTR1/5^−/−^ mouse striatum. As illustrated in Figure 3c, in R6/2 mice, NR2B in striatal neurons was expressed at cell surface, whereas in SSTR1/5^−/−^ mice, receptor-like immunoreactivity was present intracellularly. These data collectively provide comparable changes in NMDAR subunits in R6/2 and SSTR1/5^−/−^ mice with a distinct distribution pattern. As illustrated in Figure 3d, quantitative analysis revealed that R6/2 and SSTR1/5^−/−^ mice displayed 10 and 16% increase in neuronal population positive to NR1 whereas 15 and 12% increase was observed in NR2A. In contrast, neuronal population expressing NR2B declined by 21% in R6/2 mice without any significant changes in SSTR1/5^−/−^ mice brain when compared to *wt* mice.

### Receptor-specific changes in SSTR subtype in striatum of SSTR1/5^−/−^ and R6/2 mice

**Figure 4:**
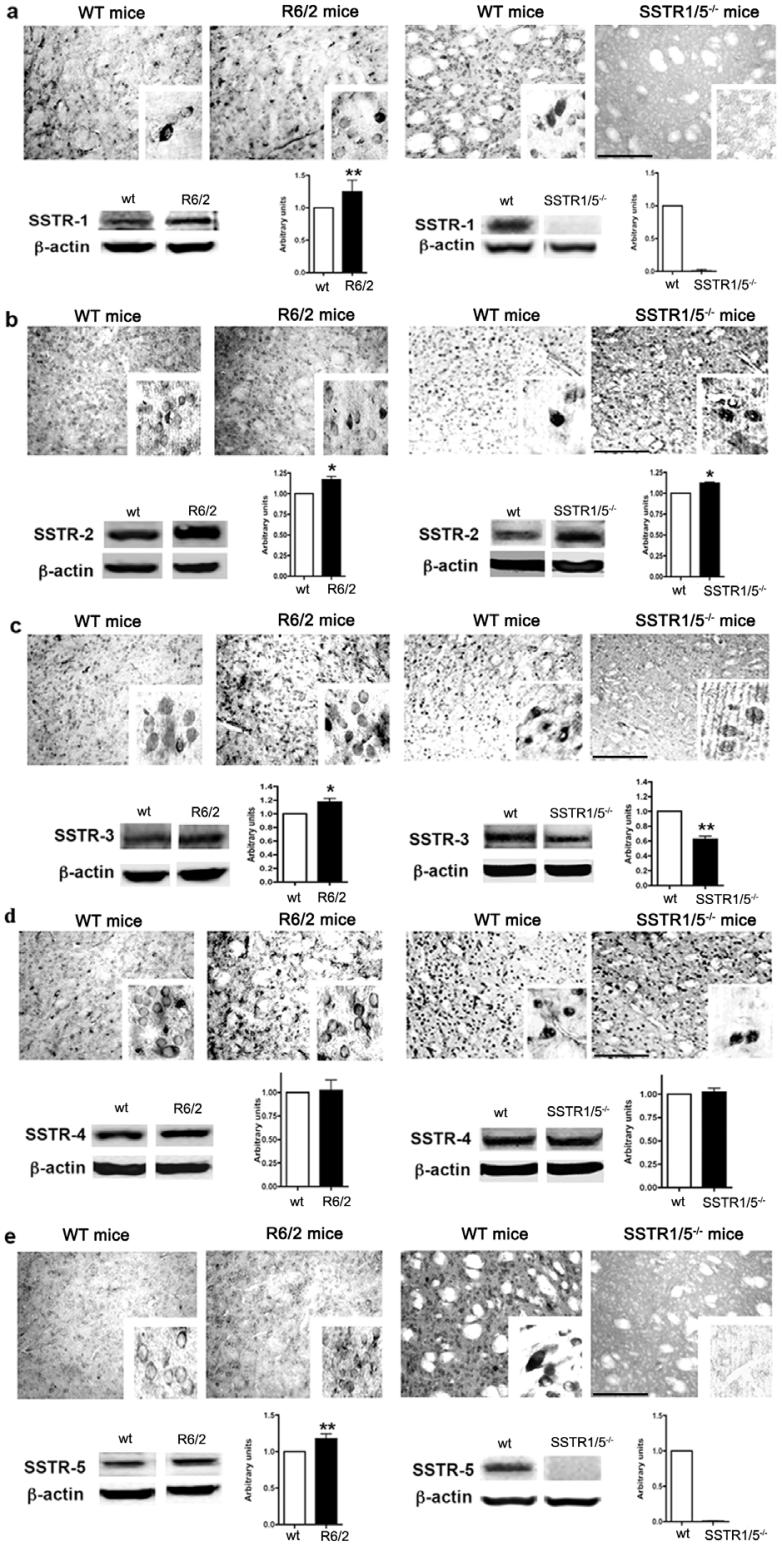
Peroxidase immunohistochemistry illustrating the changes in distribution pattern of SSTR1-5 in striatum of R6/2 and SSTR1/5^−/−^ mice. SSTR1(53 kDa) like immunoreactivity increased in striatum of R6/2 mice and abolished completely in SSTR1/5^−/−^ mice (**a**). SSTR2 (57 kDa) like immunoreactivity was increased in R6/2 and SSTR1/5^−/−^ mice (**b**). SSTR3 (60 kDa) expression was increased in R6/2 while it decreased in SSTR1/5^−/−^ mice striatum (**c**). Comparable expression of SSTR4 (44 kDa) was observed in both R6/2 and SSTR1/5^−/−^ mice brain (**d**). SSTR5 (58 kDa) expression was selectivley higher in R6/2 mice brain than *wt* and receptor like immunoreactivity was not detected in SSTR1/5^−/−^ mice (**e**). Data presented as mean ± SD (SSTR1/5^−/−^, n = 5 and R6/2 mice, n = 3) in comparison to *wt* mice brain, *P<0.05, ** P<0.01. Scale bar = 20 µm panel a–e and 5 µm for inset.

In our recent study, we have shown the expression of SSTRs in both MSNs and interneurons [45]. We have recently shown the receptor specific changes in cortical brain region of SSTR5^−/−^mice [46]. Here we describe the comparative distribution pattern of SSTR subtypes in striatal brain regions of R6/2 and SSTR1/5^−/−^ mice. In both cases, SSTRs like immunoreactivity was observed in medium-sized projection neurons, as well as in medium-sized aspiny interneurons in a receptor-specific manner. As shown in Figure 4, SSTR1-like immunoreactivity was increased in R6/2 mice in comparison to *wt,* as expected in SSTR1/5^−/−^ mice, no SSTR1 expression was detected, while *wt* mice exhibited receptor-like immunoreactivity (Figure 4a). Like SSTR1, in R6/2 and SSTR1/5^−/−^ mice striatum, SSTR2 like immunoreactivity was increased as determined by immunohistochemistry and Western blot analysis (Figure 4b). Interestingly, SSTR3 like immunoreactivity in R6/2 and SSTR1/5^−/−^ mice exhibited distinct distribution pattern. As illustrated in Figure 4c, increased expression of SSTR3 was seen in R6/2 mice in comparison to *wt*, whereas SSTR1/5^−/−^ displayed significant loss in SSTR3 likes immunoreactivity when compared with *wt*. Conversely, SSTR4-like immunoreactivity was comparable in both R6/2 and SSTR1/5^−/−^ mice without any discernable changes (Figure 4d). R6/2 transgenic mice exhibited increased expression of SSTR5 like immunoreactivity when compared with *wt* (Figure 4e). Although no SSTR5 expression was seen in SSTR1/5^−/−^ mice, SSTR5 like receptor expression was well expressed in *wt*. As illustrated in supplemental Figure S1, quantitative analysis of SSTR subtypes indicates receptor specific changes in R6/2 and SSTR1/5^−/−^. In R6/2 transgenic mice SSTR1, SSTR2, SSTR3 and SSTR5 positive immunoreactive neurons were increased by 25, 18, 19 and 17%, respectively, in comparison to *wt*. Conversely, SSTR4 positive neurons were not changed significantly in R6/2 and SSTR1/5^−/−^ in comparison to *wt.* However, in SSTR1/5^−/−^ mice SSTR2-positive neurons increased by 25%, whereas SSTR3 positive neurons declined by 38% when compared to *wt*.

### Comparable changes in signaling cascades in striatum of SSTR1/5^−/−^ and R6/2 mice

The physiological response of neurons upon activation or inhibition of GPCR or ionotropic receptors is intimately associated with the modulation of signaling pathways. Whether the ablation of SSTR modulates signaling cascades similar to the model of neurodegenerative disease has not been studied yet. Several previous studies have shown significant changes in mitogen activated protein kinases (MAPK); extracellular regulated kinase (ERK1/2) and several other key regulators associated with neuronal degeneration and pathogenesis of neurodegenerative diseases. To ascertain the underlying mechanism and functional consequences of the changes seen in SSTR and NMDAR subtypes here we determined the expression levels of pERK1/2, PKC-α, PI3K, calcineurin, calpain, synapsin-IIa, and enkephalin in striatum of R6/2 and SSTR1/5^−/−^ mice brain by Western blot analysis. As shown in Figure S2a, the expression levels of pERK1/2 were not altered in R6/2 mice whereas the status of pERK1/2 was increased in SSTR1/5^−/−^ mice but not significantly when compared to *wt*. The expression level of cell survival pathway PI3K was increased in SSTR1/5^−/−^ mice, whereas, it decreased significantly in R6/2 transgenic mice (Supplemental Figure S2b). In contrast, decreased expression of PKC-α (Figure S2c) was observed in SSTR1/5^−/−^ mice and R6/2 mice when compared to *wt*.

**Figure 5:**
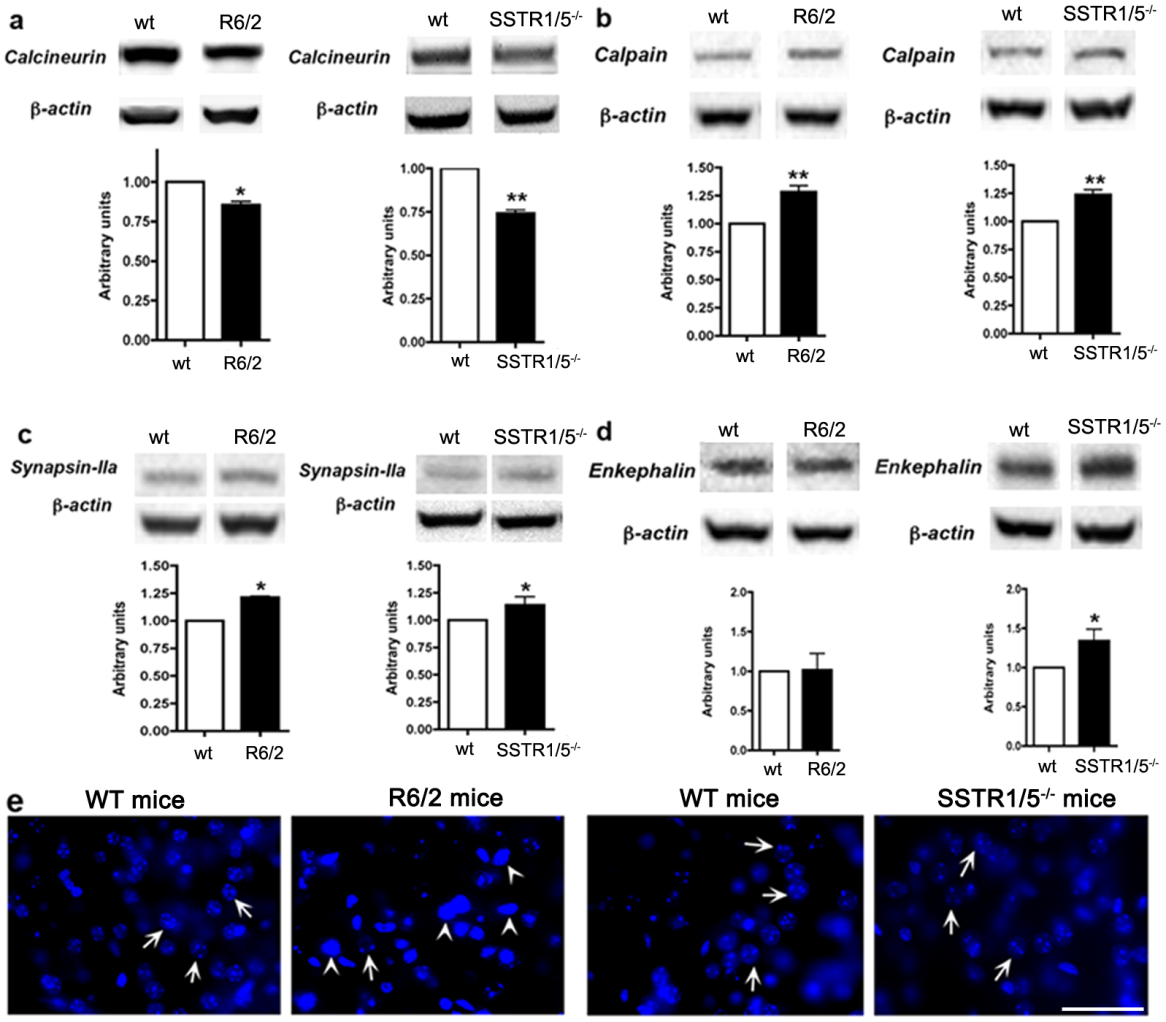
Western blot analysis demonstrating the changes in expression of calcineurin (61kDa), calpain (90 kDa), synapsin-IIa (74 kDa) and enkephalin (55 kDa) in tissue lysate prepared from striatum R6/2 and SSTR1/5^−/−^ mice. Note the loss of calcineurin in SSTR1/5^−/−^ and R6/2 mice brain striatum (**a**) and increased expression of calpain and synapsin-IIa (**b,c**). In contrast enkephalin was not changed in R6/2 mice whereas increased in SSTR1/5^−/−^ mice brain (**d**). Data presented as mean ± SD (SSTR1/5^−/−^, n = 5 and R6/2 mice, n = 3) in comparison to *wt* mice brain. *P<0.05, ** P<0.01. e) Photomicrographs showing apoptosis in R6/2 and SSTR1/5^−/−^ mice striatum. R6/2 transgenic mice displayed significantly increased number of apoptotic neuronal cells in striatum when compared to *wt* **(e panel left)**. Unlike R6/2 mice, only few apoptotic neuronal cells were observed in SSTR1/5^−/−^ mice **(e panel right)**. Arrows represent surviving cells and arrowheads represent apoptotic cells. Scale bar = 20 µm.

Previous studies have shown that the inhibitors of calcineurin accelerate HD neurological phenotype in R6/2 mice [47]. Accordingly, we determined the expression levels of calcineurin in R6/2 and SSTR1/5^−/−^ mouse brains. As illustrated in Figure 5a, calcineurin expression decreased in R6/2 and SSTR1/5^−/−^ mice, when compared to *wt* mice. Studies using YAC transgenic mice models have shown increased calpain expression, which is involved in neuronal apoptosis [48]. However, no data is presently available for calpain expression in R6/2 mice. As shown in Figure 5b, increased expression of calpain in R6/2 mice as well as in SSTR1/5^−/−^ mice was observed. Consistent with previous studies, these data strongly suggest the possible interaction between activation of NMDARs and increased expression of calpain in YAC and R6/2 transgenic mice.

Decreased expression of synapsin-IIa mRNA in striatum of R6/2 mice without significant changes in protein expression has been described earlier [43]. In further extension to our study, we next determined the expression level of synapsin-IIa and enkephalin in striatum of R6/2 and SSTR1/5^−/−^ mice and compared with *wt* using Western blot analysis. As shown in Figure 5c, synapsin-IIa expression was increased in both R6/2 and SSTR1/5^−/−^ mice, whereas, no significant change in enkephalin protein expression was observed in R6/2. However, increased expression was seen in SSTR1/5^−/−^ mice striatum in comparison to *wt* (Figure 5d).

In HD transgenic mice, namely YAC and R6/2, increased apoptosis has been described in brain due to the toxic effect of mutated Htt [49]. Here we determined whether occurrence of apoptosis is comparable in R6/2 and SSTR1/5^−/−^ mice using Hoechst 33258 staining. As shown in Figure 5e, in the absence of SSTR1/5 no apoptosis was observed. In comparison, R6/2 mice exhibited increased number of apoptotic cells when compared to their respective *wt*. These data strongly suggest that lack of SSTR1/5 does not induce excitotoxicity and the changes seen in several markers might be associated with the physiological and pharmacological effects of SSTR subtypes.

## Discussion

Receptor specific ablation of SSTR subtypes have shown behavioral changes, impaired cognitive function, loss of analgesic effect and sustained pain in a receptor-specific manner [50], [51]. Whether SSTR *ko* or any other GPCRs *ko* mice mimic the neurochemical changes similar to the neurodegenerative disorders has not been studied yet. In the present study, we compared the expression levels of key molecular markers that exert a determining role in the pathogenesis of HD in SSTR1/5^−/−^ and R6/2 mice striatum. We demonstrate that, SSTR1/5^−/−^ and R6/2 mice display comparable changes in distribution pattern of SST, NMDA receptor (NR1, NR2A and NR2B), DARPP-32, calbindin and bNOS, as well as the downstream signaling pathways including pERK1/2, calpain, synapsin IIa, PKC-α, and calcineurin in striatum. In contrast, SSTR subtypes, enkephalin, and PI3K are modulated in strain specific manner. This is the first comprehensive description showing comparable neurochemical and biochemical changes in a transgenic model of HD and SSTR1/5^−/−^ mice brains.

The most coherent finding in HD pathology as well as in transgenic mice is the loss of DARPP-32 [43]. Consistent with this study, we describe comparable changes in DARPP-32 like immunoreactivity in R6/2 and SSTR1/5^−/−^ mice, exhibiting >60% loss of DARPP-32. For the first time, we have demonstrated a similar pattern of DARPP-32 loss in the striatum of SSTR1/5 ablated mice and HD transgenic mice R6/2 indicating that SSTRs might be involved in regulation of DARPP-32. The DARPP-32 mediated downstream signaling pathway is modulated by dopamine receptors (DRs), another prominent member of GPCR family [52],[53]. SSTRs share ∼30% structural homology with DRs and constitute functional heterodimers with distinct pharmacological properties and enhanced signaling [36], [54]. In agreement with this information, SSTRs, directly or indirectly, via interaction with DR, may modulate DARPP-32. This concept is further strengthened by our recent studies showing receptor- and region-specific colocalization of DARPP-32 and DRs in rat brain [55]. DARPP-32 becomes phosphorylated in response to the activation of D1R [43]. Conversely, D2R activation inhibits DARPP-32 phosphorylation. Whether SSTR subtype regulates DARPP-32 phosphorylation is not known. Here we provide the evidence that in the absence of SSTR subtypes, DARPP-32 expression is decreased in striatum in a similar manner as seen in HD brain and/or HD transgenic mice. The loss of DARPP-32 like immunoreactivity in SSTR1/5^−/−^ mice is not surprising; as previous studies have also shown that DARPP-32^−/−^ mice resemble HD mice in dopamine signaling [43].

Consistent with the existing notion that bNOS positive neurons are preserved in HD, the expression pattern and quantification of bNOS positive neurons in R6/2 mice as well as SSTR1/5^−/−^ mice is comparable. Similar is the pattern of SST expression in both strains. In line with previous observations, our results further strengthen the concept of selective preservation of medium-sized aspiny interneurons expressing SST/b-NOS [8]. Furthermore, our previous studies have shown that blockade of SST using antisense oligonucleotide leads to the loss of bNOS/NADPH-d positive neurons upon QUIN/NMDA treatment [31]. Taken together, these results support the notion that the presence of SST is likely responsible for the survival of aspiny interneurons in excitotoxicity.

In HD, the activation of NMDAR is one of the leading causes of neuronal loss, in addition to the mutation in Htt. Functional and physiological significance of NMDARs has recently been described in pathophysiology of HD and reported that the NMDAR antagonist, memantine, blocks the nuclear inclusion of mutated Htt seen in HD [19]. Furthermore, recent studies have shown the distinct role of synaptic and extrasynaptic NMDARs in early and late onset of HD [56], [57], [58]. NMDAR positive neurons are most vulnerable in HD as well as in various mouse models of excitotoxicity [18], [59], [60], [61]. Cumulatively, these studies indicate an increase NR1 and the loss of NR2B expression. However, studies for NR2A are controversial and such discrepancies may be due to the mouse strain used as a model. In the current study, an increased NR1 and NR2A expression, with the loss of NR2B immunoreactivity in both R6/2 and SSTR1/5^−/−^ mice may be linked to the neurodegeneration of MSNs, which is attributed to an increased Ca^2+^ influx. Moreover, in SSTR1/5^−/−^ mice, NR2A and NR2B immunoreactivity accumulates intracellularly while in R6/2 both receptors are well expressed at cell surface. In light of these results, we propose two different mechanisms for the role in NMDAR-mediated neurotoxicity. First, the membrane expression of NR2A and NR2B in HD transgenic mice allows receptor interaction at cell surface, which results in excitotoxicity. Consistent with the existing concept that NMDARs are functionally active in heteromeric complex, the increased cell surface expression of NR1 and NR2A in R6/2 might be involved in excitotoxicity through heterodimerization. In the absence of SSTR subtypes, NMDAR trafficking might be impaired, leading to receptor accumulation intracellularly. Whether SSTR and NMDAR functionally interact with each other is not known and further studies are in progress to determine this. Studies are warranted to delineate the molecular mechanism for the intracellular accumulation of NMDAR in SSTR1/5^−/−^ mice. Furthermore, the possibility of impaired mitochondrial function in these processes cannot be ruled out.

The physiological response of cells upon the activation of SSTRs is receptor specific and can display multiple effects. SSTR2 is known to inhibit Ca^2+^ activated channels and increased neuronal Ca^2+^ is detrimental in excitotoxicity *in vitro* as well as in HD. Our results show increased expression of SSTR2 in the absence of SSTR1/5 and concomitantly in HD transgenic mice, suggesting a compensatory mechanism to inhibit Ca^2+^ due to enhanced excitatory input via the activation of NMDAR. In support, we have recently shown that SSTR2 and SSTR5 heterodimerize with significant changes in receptor pharmacological properties as well as enhanced signaling [62]. Furthermore, SSTR1/5^−/−^ mice exhibit increased expression of D2R in comparison to *wt* (unpublished observations). Since SSTR5 and D2R functionally interact and exist in a heteromeric complex, the increased expression of D2R in SSTR1/5^−/−^ mice supports the compensatory role in the absence of SSTR5 *in vivo* [36].

Increased expression of NR1 and NR2A might be linked with the decreased expression of calcineurin and increased expression of calpain in R6/2 and SSTR1/5^−/−^ mice. Calcineurin is involved in the phosphorylation of DARPP-32, which further regulates the cell survival pathways. Calcineurin knockout mice show the inhibition of motor functions, loss of synaptic plasticity, learning and memory [63]. The loss of calcineurin expression in R6/2 and SSTR1/5^−/−^ mice might correlate with the symptoms of HD. Furthermore, the loss of calcineurin and DARPP-32 expression can be correlated with the decreased expression of PKC-α. PKC-α plays a role in regulation of membrane associated signal transduction pathways mediated by Ca^2+^ homeostasis [64]. DARPP-32 phosphorylated at Thr34 (threonine) via PKC-α converts into protein phosphatase-1 (PPtase-1), which in turn inhibits the phosphorylated forms of calcium response element binding protein (CREB) and leads to cell survival [65]. Alternatively, the loss of PKC-α expression can also be correlated with increased apoptosis detected in R6/2 mice. However, decreased expression of PKC-α may also be associated with decreased expression of DARPP-32 in R6/2 and SSTR1/5^−/−^ mice. Changes in pERK1/2 can be correlated with the variable expression of SSTRs. Increased pERK1/2 in SSTR1/5^−/−^ mice may be due to the loss of SSTR5, which is known to inhibit pERK1/2 and increased expression of SSTR5 in R6/2 might exert inhibitory role on pERK1/2.

Previous studies have shown increased calpain activity in HD human brain tissue and YAC transgenic mice [48], [66], [67]. Increased expressions of calpain in R6/2 and SSTR1/5^−/−^ mice further suggest a role of NMDAR-mediated cell death [48]. Enkephalin primarily functions as an anti-nociceptive but is also disrupted in early-onset of HD [68]. In agreement with previous studies, we detected no changes in enkephalin expression in R6/2 mice [68]. However, these results contradict other findings, which report a decrease in enkephalin expression [69]. Such discrepancies may be due to the isoform of enkephalin targeted. In SSTR1/5^−/−^ mice, increased expression of enkephalin, in part, may be indirectly due to changes in opioid receptors, since SSTRs share 40% structural homology and exhibit heterodimerization [37].

Post-mortem brains from HD patients and transgenic mice have shown augmented apoptosis in striatum [18]. Amongst all SSTRs, SSTR3 is the only known receptor subtype that can induce apoptosis [33]. Whether increased expression of SSTR3 as seen in R6/2 mice is associated with apoptosis in HD needs further investigation. However, decreased SSTR3 expression in SSTR1/5^−/−^ mice may account, albeit to a lesser degree, for apoptotic cell death in comparison to R6/2. SST via activation of SSTR subtypes blocks Ca^2+^ influx through the interference with NMDA function. This suggests that mice lacking SSTR1/5 may cause pronounced NMDA-induced toxicity due to the activation of NMDA receptors in SSTR1/5^−/−^ mice. This speculation is consistent with previous studies from HD transgenic mice [18]. Furthermore, the inhibitory effect of SST on excitatory synaptic transmission is also well established in rodent brain [33].

Previous studies have shown that presymptomatic HD mice (6–8 weeks old R6/2 mice) not only display physiological characteristics but also behavioral and neurochemcial changes similar to those observed in HD [70]. These observations indicate that SSTR1/5^−/−^ might serve as a model to elucidate the role of SSTR subtypes to characterize the neurochemcial changes in different proteins and pathways involved in HD. SSTR5 blocks the L-type Ca^2+^ channels and may correlate with the increased expression of SSTR5 in R6/2 mice [71]. As SSTR5 is not predominantly expressed in mouse striatum and SSTR1 and SSTR5 are known to form heterodimers, we speculate that the increased expression of SSTR1 in R6/2 mice may be due to a compensatory mechanism of SSTR5. Our observations raise a potential question about the role of SSTR1 or SSTR5 independently. We did not eliminate this question from our discussion and propose that knocking out SSTR1 or SSTR5 might have same effect as seen in double *ko* mice, albeit to the lesser degree. We have recently characterized the SSTR subtypes for homo-and heterodimerization in detail with distinct pharmacological and enhanced functional properties [34], [35], [72].

Taken together, our results indicate that SST and SSTRs might play an important neuroprotective role in neurodegenerative diseases. Targeting this pathway can provide novel insights in understanding the pathophysiology of neurodegenerative diseases. This is the first study showing similar disease characteristics related to neurochemical changes upon ablation of the GPCR gene (i.e., SSTRs). We here reinforced the concept that mice in the absence of SSTR subtype might have greater susceptibility to neurodegenerative diseases as well as excitotoxicity. We propose that in HD transgenic mice as well as in HD patient's pharmacological restoration and maintenance of somatostatinergic system might serve as a beneficial therapeutic approach in treatment of HD.

## Materials and Methods

### Materials

Rabbit polyclonal antibodies for SSTR subtypes have been previously characterized [42], [45], [73]. Mouse monoclonal antibodies for DARPP-32 and calcineurin were purchased from BD Biosciences (Mississauga, ON, Canada). Antibodies against NMDAR-1, NMDAR-2A, and NMDAR-2B were obtained from Millipore (CA, USA) and anti-calbindin D-28K from Sigma-Aldrich (MO, USA). PKC-α, ERK1/2, synapsin-IIa and Met/Liu enkephalin were obtained from Cell Signaling Technology (MA, USA). The peroxidase vectastain ABC kit was purchased from Vector Laboratories (CA, USA). Nitrocellulose Hy-bond membrane and enhanced chemiluminescence (ECL) detection kit was obtained from Amarsham Ltd. (Oakdale, ON, Canada). All other chemicals of analytical grade were obtained from various commercial sources.

### Animals

Perfused and frozen brains from 8-week old HD (C57BL/6 X CBA)F1(B6CBA)-TgN (HDexon1)62 (R6/2) carrying 120 +/− 5 CAG repeat expansions and [wild-type(*WT*)]B6CBAF_1_/J mice were obtained from Jackson Laboratory (Sacramento, CA, USA). Similarly, frozen and perfused brains from 8 weeks old SSTR1/5^−/−^ mice and *wt* were kindly provided by Dr. F. C. Brunicardi (Michael E. DeBakey Department of Surgery, Baylor College of Medicine, Houston, Texas, USA). Further details for the creation and characterization of SSTR1/5^−/−^ mice have been described in detail previously [74]. The protocols regarding animal care were followed in compliance with the Institute of Laboratory Animal Resources, Commission on Life Sciences, National Research Council. The *wt* and SSTR1/5^−/−^ mice were housed at Baylor college of Medicine, Houston, Texas, USA with 12-hour light/dark cycle.

### Immunohistochemistry studies

In the present study, three male adult mice from each group, *wt* and R6/2 and five male adult mice from *wt* and SSTR1/5^−/−^ mice were used. 30 µm thick coronal brain sections were cut on Leica vibratome. Immunostaining was performed on free-floating sections using the avidin–biotin peroxidase Vectastain ABC kit as described previously [45], [73], [75]. Briefly, brain sections were incubated with 0.3% hydrogen peroxide followed by incubation with 0.02% Triton X-100. Following three washes, sections were incubated with 5% NGS for 1 h at room temperature (RT) and followed by overnight incubation with specific primary antibodies against rabbit-anti-SSTR1-5 (1∶500), rabbit-anti-SST (1∶600), mouse anti-DARPP-32 (1∶250), mouse anti-NMDAR-1, NMDAR-2A, NMDAR-2B and calbindin D-28K (1∶500) and rabbit anti-bNOS (1∶400) in 1% NGS at 4°C in humid atmosphere. The sections were incubated with the biotinylated secondary antibodies; goat-anti-rabbit to detect SSTRs, SST, and bNOS and goat anti-mouse to detect DARPP-32, calbindin and NMDARs and followed by incubation in ABC complex for 30 min. The final color was developed by adding a mixture containing 0.001% hydrogen peroxide and 0.2 mg/ml of 3,3-diaminobenzidine (DAB) for 3–4 min in 50 mM Tris buffer. Sections were washed in PBS, mounted onto slides and viewed and photographed under Leica microscope attached with the Retiga 2000R camera.

### Western blot analysis

Striatal tissues isolated from R6/2 or SSTR1/5^−/−^ mice and respective *wt* brains were homogenized in homogenizing buffer containing (62.5 mM Tris-HCl, 50 mM dithiothretiol [DTT], 10% glycerol, 2% SDS). 15 µg of total protein was fractioned by electrophoresis on 10% SDS polyacrylamide gel, transferred onto 0.2 µm-nitrocellulose membrane. The membrane was blocked with 5% non-fat dried milk at RT for 1 h and incubated overnight at 4°C with specific primary antibodies against– SSTRs1-5, NMDARs, bNOS (1∶600), SST, enkephalin, synapsinIIa, calpain, calcineurin, calbindin, phospho and total ERK1/2, PI3K (1∶1000), DARPP-32 (1∶250) and PKC-α (1∶500). Following three subsequent washes, the membrane was incubated with peroxidase conjugated goat anti-rabbit or a goat anti-mouse secondary antibody at RT for 1 h. The bands were detected using chemiluminescence reagent and images were taken using an Alpha Innotech FluorChem 8800 (Alpha Innotech Co., San Leandro, CA) gel box imager. β-actin was used as the housekeeping protein for loading control. The bands intensity was quantified using densitometric analysis and the changes in protein expression were calculated as the ratio of band of interest and the density of β-actin.

### Quantitative analysis

Quantitative analysis on brain sections was performed using NIH Image J software as described earlier [45]. Neurons were considered immunoreactive if the labeling of their cell bodies was distinctly higher than the background staining obtained in the presence of pre-absorbed or in the absence of primary antibodies. For quantitative analysis five mice were taken from *wt*, SSTR1/5^−/−^ mice and three for *wt* and R6/2 mice. 12–15 randomly selected areas from 6–7 sections from individual mouse brain were used for neuronal counting. Since our immunohistochemical studies provide semiquantitative analysis of immunoreactivity in R6/2 or SSTR1/5^−/−^ and respective *wt* mice we took all the possible precaution to keep our experimental conditions consistent in all aspects, i.e., incubation timings with antibodies (18 h) and 3, 3-diaminobenzidine for the final color development (4 mins).

### Statistical analysis

Data are presented as percentage changes in neuronal population for positive immunoreactivity from 6–7 brain sections per mice. Total of (6000–6500) neurons were counted in each condition. Bars represent the mean ± SEM (n = 3 for R6/2 and n = 5 for SSTR1/5^−/−^). Statistical comparison between *wt* and R62 (n = 3) and *wt* and SSTR1/5^−/−^ (n = 5) mice were analyzed using student t-test (*p<0.05). GraphPad Prism 5.0 (San Diego, CA) was used to perform all the statistical analysis.

## Supporting Information

Figure S1 Quantitative analysis of SSTR postive neurons for receptor specific changes in SSTR1/5^−/−^ and R6/2 mice strains. Note receptor specific changes in the numbers of SSTR1–5 positive neurons in SSTR1/5^−/−^and R6/2 mice compared to the respective *wt*. Data presented as mean ± SD for neuronal quantification (SSTR1/5^−/−^, n = 5 and R6/2 mice, n = 3) in comparison to *wt* mice brain, *P<0.05, ** P<0.01.
(TIF)

Figure S2 Downstream signalling pathways are differentialy regulated in R6/2 and SSTR1/5^−/−^ mice. Tissue lysate prepared from striatum of R6/2 and SSTR1/5^−/−^ mice brain was fractionated on 10% SDS PAGE and membrane was blotted for total and phosphorylated ERK1/2 and PI3K and PKC-α. The status of p-ERK1/2 is not changed in R6/2 and SSTR1/5^−/−^ mice striatum (**a**). Note the decreased expression level of PI3K (110 kDa) in R6/2 mice whereas SSTR1/5^−/−^ mice brain exhibited increased PI3K expression (**b**). In contrast PKC-α (80 kDa) decreased in R6/2 and SSTR1/5^−/−^ mice brain (**c**). Data presented as mean ± SD (SSTR1/5^−/−^, n = 5 and R6/2 mice, n = 3) in comparison to *wt* mice brain, *P<0.05, ** P<0.01, ***P<0.001.
(TIF)
