# Supplementary figures and images for: Somatostatin Receptor 1 and 5 Double Knockout Mice Mimic Neurochemical Changes of Huntington's Disease Transgenic Mice
Source: PLoS One. 2011 Sep 2;6(9):e24467. doi: 10.1371/journal.pone.0024467 (PMC3166321; doi:10.1371/journal.pone.0024467)

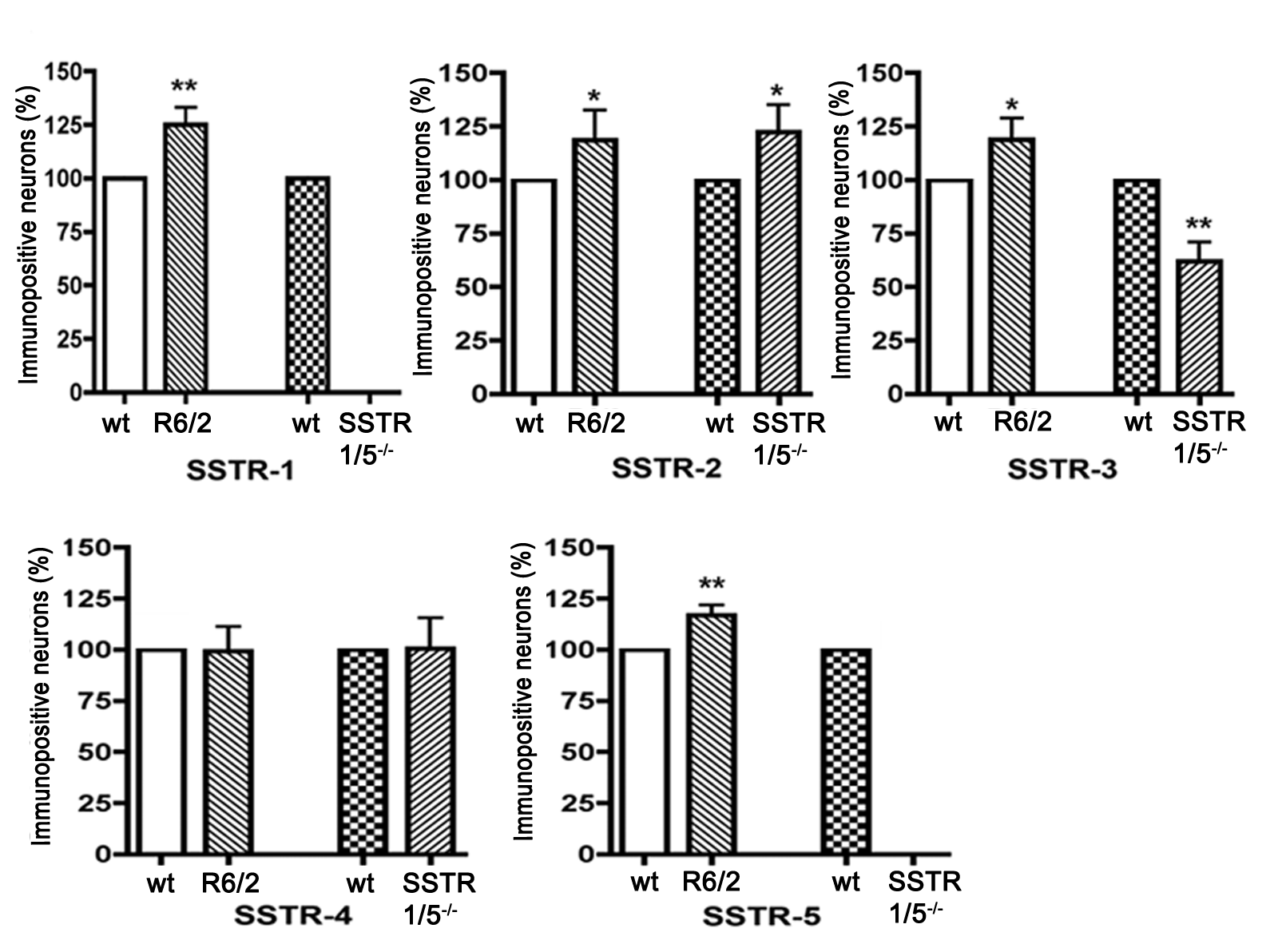

Supplement: Figure S1 — Quantitative analysis of SSTR postive neurons for receptor specific changes in SSTR1/5−/− and R6/2 mice strains. Note receptor specific changes in the numbers of SSTR1–5 positive neurons in SSTR1/5−/−and R6/2 mice compared to the respective wt. Data presented as mean ± SD for neuronal quantification (SSTR1/5−/−, n = 5 and R6/2 mice, n = 3) in comparison to wt mice brain, *P<0.05, ** P<0.01. (TIF) [file pone.0024467.s001.tif]

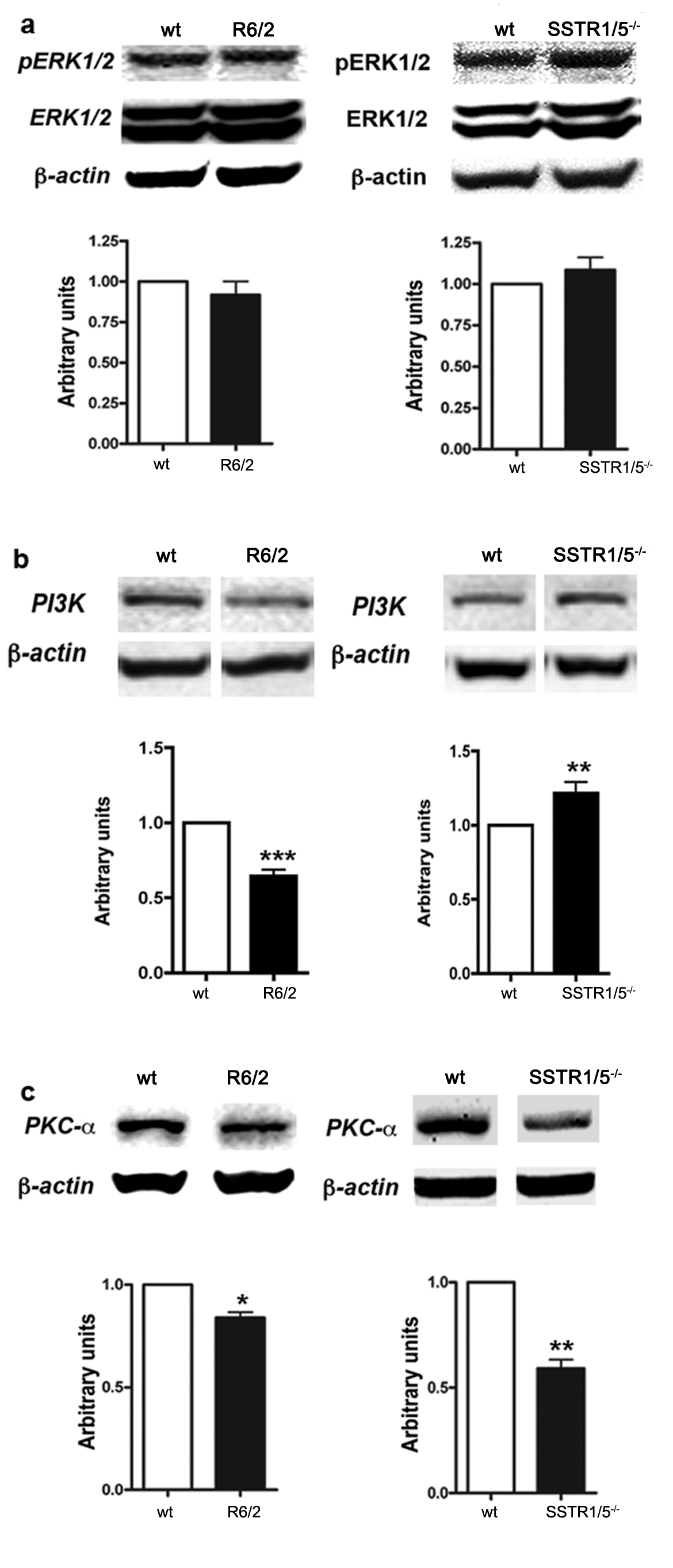

Supplement: Figure S2 — Downstream signalling pathways are differentialy regulated in R6/2 and SSTR1/5−/− mice. Tissue lysate prepared from striatum of R6/2 and SSTR1/5−/− mice brain was fractionated on 10% SDS PAGE and membrane was blotted for total and phosphorylated ERK1/2 and PI3K and PKC-α. The status of p-ERK1/2 is not changed in R6/2 and SSTR1/5−/− mice striatum (a). Note the decreased expression level of PI3K (110 kDa) in R6/2 mice whereas SSTR1/5−/− mice brain exhibited increased PI3K expression (b). In contrast PKC-α (80 kDa) decreased in R6/2 and SSTR1/5−/− mice brain (c). Data presented as mean ± SD (SSTR1/5−/−, n = 5 and R6/2 mice, n = 3) in comparison to wt mice brain, *P<0.05, ** P<0.01, ***P<0.001. (TIF) [file pone.0024467.s002.tif]
